# Food Habits in Atopic Patients in Iranian Children

**Published:** 2012-07-30

**Authors:** M H Bemanian, Sh Hashemzadegan, M Nabavi, M Rezaeisadrabadi

**Affiliations:** 1 Department of Pediatrics, Shahid Sadoughi University of Medical Sciences, Yazd, Iran; 2 Asthma and Allergy Research Institute, Tehran University of Medical Sciences, Tehran, Iran; 3 Faculty of Medicine, Shahid Sadoughi University of Medical Sciences, Yazd, Iran; 4 Faculty of Medicine, Hazrat-e-Rasoul Hospital, Tehran University of Medical Sciences, Tehran, Iran

**Keywords:** Allergy, Food, Asthma

Dear Editor,

The prevalence of allergic diseases has been increased during recent years; especially in industrialized countries.[1] The relationship between allergic disease and genetic origins was proved while some investigations considered environmental factors as an important cause of these diseases. It seems that alteration in life style and food habit have played more important role in prevalence of allergic diseases.[2]

It was reported that food may have an important role to develop asthma because of epigenetic effects,[3] thus increasing in the prevalence of asthma and allergy that can be related to dietary habits.[4] Allergic reactions induced by food may involve different tissues like skin and respiratory tract.[5] Relationship between asthma and some kinds of foods like starch, cereal and vegetable consumption was noticed in the International Study on Allergies and Asthma in Childhood (ISAAC).[6]

In a recent survey, 110 cases with mean age of 9.1 years old and 110 controls with mean age of 10.2 years old were studied. They had a mean weight of 28.7 and 35.2 kilograms respectively. Both of case and control groups were compared by diet habit questionnaire including fast foods, potato chips, chocolate and popcorn.

According to our data, height and weight of case group was less than the control group that may be due to consumption of chocolate, popcorn and food additives or may be due to an underlying disease. It was shown that pizza (9.1%), chocolate (8.6%), salami (5.5%) and popcorn (3.6%) were respectively the most causative agents for presence of symptoms of atopic disease in our area. It was similar to Wickens et al. research in which they concluded that fast food resulted into an increase in BMI and bronchial hyper-responsiveness (BHR).[7] This difference may be due to the kind of fast food and other food allergens. An investigation in Hastings, New Zealand demonstrated that hamburger in children may be a cause of development of asthma. In our study, chocolate consumption was significantly more than the control group. This agrees with results of Cohen et al. who found a correlation between different kinds of chocolate and atopic disease in children.[8]

There was a significant difference between case and control group in consumption of popcorn and potato chips. Sahakian et al. believed that this effect was related to flavoring chemicals used in these products.[9] According to a similar study performed in Spain, tree nut was shown to increase allergic symptoms in patients, but this effect was not seen in our survey.[10]

According to our data, height and weight of case group was less than the control group while they consumed more chocolate and pizza. The presence of food additives and presence of an underlying disease may explain the presence of more symptoms of atopic disease among them which can be of public health importance.
